# Pulmonary cryptococcosis masquerading as lung metastasis in gynecologic cancers: Two case reports

**DOI:** 10.1097/MD.0000000000036274

**Published:** 2023-11-24

**Authors:** Seul Yi Lee, Yong Jung Song, Geewon Lee, Hyung Joon Yoon, Kyung Un Choi, Dong Soo Suh, Ki Hyung Kim

**Affiliations:** a Department of Obstetrics and Gynecology, Pusan National University School of Medicine, Busan, Republic of Korea; b Research Institute for Convergence of Biomedical Science and Technology, Pusan National University Yangsan Hospital, Yangsan, Republic of Korea; c Department of Radiology, Pusan National University School of Medicine, Busan, Republic of Korea; d Department of Pathology, Pusan National University School of Medicine, Busan, Republic of Korea; e Biomedical Research Institute, Pusan National University Hospital, Busan, Republic of Korea.

**Keywords:** gynecological cancer, lung metastasis, misdiagnosis, pulmonary cryptococcosis

## Abstract

**Rationale:**

Pulmonary cryptococcal infections occur mainly in immunocompromised individuals, such as those with malignancies. Preoperative diagnosis of pulmonary cryptococcosis (PC) can be challenging for both clinicians and radiologists because of nonspecific clinical manifestations and variable radiologic features, as it is easily misdiagnosed as metastatic lung cancer.

**Patient concerns:**

In case 1, a 76-year-old woman with a history of cervical cancer presented with lung nodules detected on chest computed tomography (CT) 13 months after completing concurrent chemoradiotherapy. In case 2, a 56-year-old woman with a history of ovarian cancer presented with pulmonary nodules on chest CT 19 months after completing chemotherapy. Both patients were clinically asymptomatic, and tumor markers were not elevated.

**Diagnoses:**

In case 1, chest CT revealed multiple enhanced nodules with lobulated margins in the left lower lobe, and positron emission tomography (PET)-CT showed uptake in the nodule with a standardized uptake value of 3.7. In case 2, chest CT revealed several nodules in the right upper lobe abutting the right major fissure, and PET-CT revealed fluorodeoxyglucose uptake in the nodules. Pathology revealed granulomatous inflammation with cryptococcal infection, and mucicarmine and periodic acid-Schiff staining confirmed cryptococcal infection in both cases.

**Interventions:**

Presumptive diagnoses of lung metastases were made in both cases and thoracoscopic lobectomy was performed. Postoperatively, the patients received antifungal therapy with fluconazole.

**Outcomes:**

PC was differentially diagnosed and effectively managed. The patients remained disease-free for both PC and gynecological cancers during subsequent follow-ups.

**Lessons:**

Recognition that PC can mimic lung metastasis is important for managing gynecological cancers. PC should be considered in the differential diagnosis when single or multiple nodules are detected on chest radiography without elevation of tumor markers in patients with gynecological cancer.

## 1. Introduction

Cryptococcal infections are commonly observed in immunocompromised patients, such as those infected with human immunodeficiency virus (HIV) or cancer.^[1,2]^ The incidence of pulmonary cryptococcosis (PC) has increased in recent years owing to its occurrence in immunocompetent patients. Furthermore, it was recently reported that 13% of HIV patients with pneumonia were positive for cryptococcal antigens.^[3]^ Malignancy and its treatment may also confer a higher risk of cryptococcal infection, and the prevalence of PC among patients with solid organ malignancy receiving chemotherapy is approximately 8%.^[4]^ However, 60% of PC cases are diagnosed in immunocompetent patients.^[4]^ PC has variable clinical features and nonspecific imaging findings; thus, differentiating PC and lung metastasis from the various lung nodules found on chest computed tomography (CT) during gynecological cancer follow-up is challenging. A nodule/mass-pattern, either solitary or multiple, with a peripheral distribution of lesions, is the most common finding in PC. Radiographic findings of PC can mimic those of lung cancer, because they form nodule-like masses in the lungs.^[5]^ Therefore, in some cases, cryptococcal infection discovered incidentally by radiologic tests routinely performed for the regular follow-up of cancer patients is misunderstood as lung metastasis. Although some characteristic CT manifestations of PC have recently been described, they are still considered nonspecific and similar to those of lung metastases.^[6,7]^ Recognition that PC can mimic lung metastasis is important for the management of patients with gynecological cancers, and accurate diagnosis and understanding of the disease are essential for appropriate management. Here, we present 2 cases of PC mimicking lung metastasis in gynecological cancer patients.

## 2. Case report

### 2.1. Case 1

**Figure 1.**
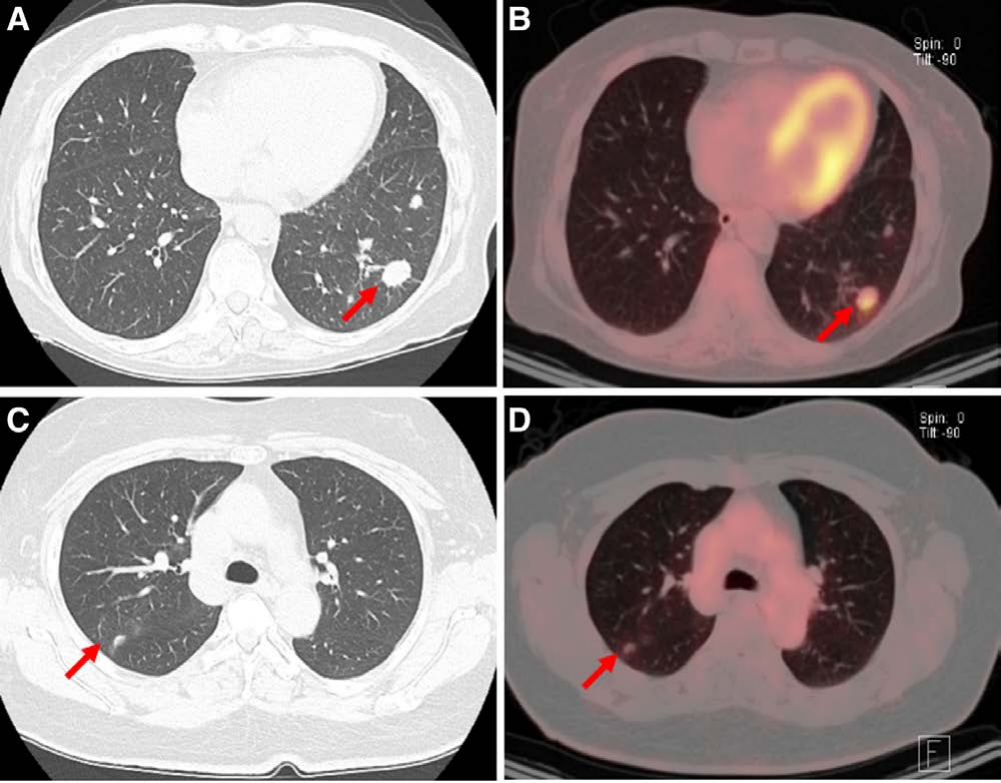
Radiological findings. Computed tomography revealing multiple nodules with lobulated margins and mild enhancement in the left lower lobe (A) and several small nodules in the right upper lobe (C). Positron emission tomography-computed tomography images demonstrating increased FDG uptake in the nodules (B and D). FDG = fluorodeoxyglucose.

**Figure 2.**
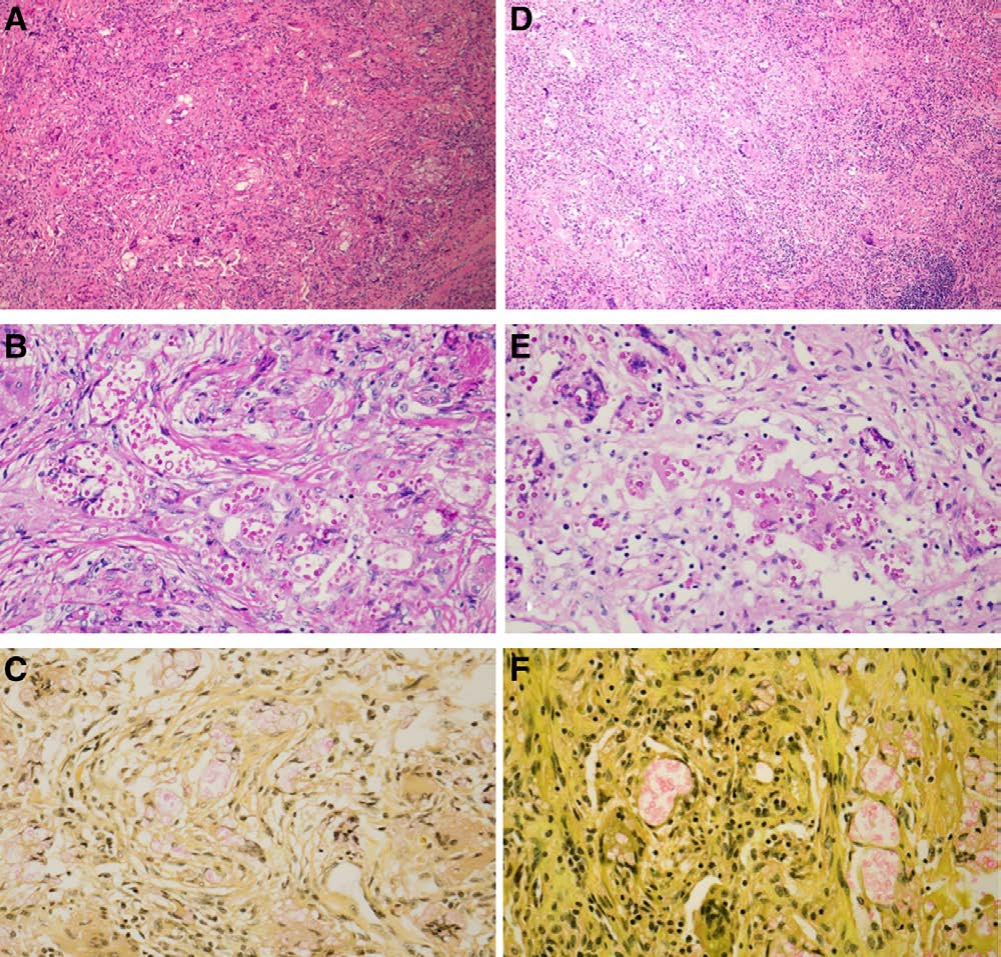
Pathological examination revealed cryptococcal infection. (A and D) H&E slides showing granulomatous inflammation (×100). (B and E) PAS stain (×400) decorating the yeasts and (C and F) mucicarmine stain (×400) decorating the characteristic gelatinous capsule a bright pink.

Routine chest CT detected lung nodules in a 76-year-old woman after 13 months of concurrent chemoradiotherapy for cervical cancer. The patient underwent a radical hysterectomy and pelvic lymphadenectomy for squamous cell carcinoma on February 18, 2021. Postoperatively, concurrent chemoradiotherapy consisting of 50.4 Gy of whole pelvis irradiation in 28 fractions and 6 weeks of cisplatin (40 mg/m^2^/week) was administered from May 13, 2021, to June 22, 2021, after which she had no respiratory symptoms, a normal serum squamous cell carcinoma level, and a negative HIV test result. However, chest CT revealed multiple nodules with lobulated margins and enhancement in the left lower lobe (Fig. 1A). The largest nodule measured 1.7 cm in diameter. Positron emission tomography (PET)-CT showed uptake in the nodules and a standardized uptake value of 3.7 (Fig. 1B). Based on the CT and PET-CT findings, a presumptive diagnosis of lung metastasis was made and video-assisted thoracoscopic left lower lobectomy was performed on October 7, 2022. The lung parenchyma was relatively healthy; however multiple nodules in the left lower lobe and visceral pleural dimpling were observed. Pathological examination revealed that the nodules were associated with fungal infections and produced no evidence of metastasis. Anthracotic pigmentation around the bronchovascular bundles, focal bronchioiloectasis, patchy atelectasis, and emphysema were observed in the subpleural areas in the adjacent lung parenchyma. Mucicarmine and periodic acid-Schiff (PAS) staining confirmed the cryptococcus infection (Fig. 2A–C). However, these specimens were negative for TTF-1 and tumor protein 63. Serum cryptococcal antigen test result was negative. The patient was administered fluconazole antifungal therapy and was disease-free at 12 months postoperatively.

### 2.2. Case 2

Routine chest CT detected pulmonary nodules in a 56-year-old woman 19 months after completing chemotherapy for ovarian cancer. She also had type 2 diabetes mellitus (DM). The patient underwent surgery on June 4, 2020, for coexisting stage III ovarian cancer and stage I endometrial cancer, which had been treated with 6 cycles of platinum-based chemotherapy (carboplatin-paclitaxel) from June 26, 2020, to October 20, 2020. The patient was clinically asymptomatic with no respiratory symptoms, in good general condition, had a normal serum CA-125 level, and was HIV-negative. Chest CT revealed several nodules in the right upper lobe abutting the right major fissure (Fig. 1C). The correlating PET-CT image revealed fluorodeoxyglucose uptake in the nodules (maximal standardized uptake value, 1.4) (Fig. 1D). Lung metastasis was considered based on the CT and PET-CT findings, and the nodules were subsequently resected via video-assisted thoracoscopic right upper lobectomy on July 21, 2022. Pathological examination revealed granulomatous inflammation with cryptococcal infection and no evidence of metastasis. Specimens stained negative for PAX-8 and ER, mucicarmine and PAS staining revealed the presence of fungal yeasts (Fig. 2D–F). Serum cryptococcal antigen test result was negative. Fluconazole was administered orally, and 13 months after the nodule resection, there was no evidence of relapse. Ethical approval was waived by the Institutional Review Board (IRB) of Pusan National University Hospital because of the retrospective nature of the study. Written informed consent was obtained from the participants for publication of details of their medical cases and accompanying images.

## 3. Discussion

We present 2 cases of PC in gynecological cancer patients with underlying medical conditions or predisposing factors that were initially misdiagnosed as lung metastasis. Cryptococcus is an encapsulated yeast with global distribution.^[8]^ PC may develop after inhaling cryptococcus spores, and mainly affects immunocompromised patients, such as those with HIV, an underlying disease (e.g., a hematologic or solid organ malignancy, DM, lung disease, autoimmune disease, or liver cirrhosis), and those who have undergone organ transplantation and are receiving corticosteroid or immunosuppressive therapy. DM is commonly associated with PC (as in case 2) because hyperglycemia impairs alveolar macrophages and immune function. Malignancy is also a leading risk factor associated with 5% to 27% of cryptococcal infections.^[9]^ Reported risk factors for PC in cancer patients include neutropenia, lymphopenia, and steroid use.^[10]^ In our study, PC developed in immunosuppressed patients with underlying medical conditions (DM) or predisposing factors (chemotherapy and radiotherapy). However, recently, healthy individuals without immunodeficiency have been reported to account for the majority (≥60%) of patients with PC.^[4,11]^

Gynecologic cancers can metastasize to the lungs, and their prognoses, prevalence, and behaviors depend on cancer type. Of the patients with stage IV gynecological cancers registered in the SEER database from 2010 to 2015, 42%, 40%, and 18% originated from ovarian, uterine, and cervical cancers^[12]^ and the percentages of these cancers that metastasized to the lungs were 38%, 62%, and 59%, respectively.

The clinical manifestations of cryptococcal infections are variable and range from asymptomatic to life-threatening. Approximately 51.7% of patients with PC have no clinical symptoms and are diagnosed incidentally during routine health screenings.^[7]^ Furthermore, it is difficult to differentiate PC from metastatic lung cancer with multiple nodules. Consequently, PC is often misdiagnosed as metastatic lung cancer because of its similar clinical manifestations and radiological characteristics. In a report on the misdiagnosis of PC, the rate of misdiagnosis was 85.4%,^[13]^ and of these misdiagnoses, 36.7% were misdiagnosed as pneumonia, 31.7% as lung cancer, and 17.1% as tuberculosis.

PC can be diagnosed based on culture, serum antigen testing, radiographic findings, and histopathological examinations. The sputum, bronchial lavage, and pleural effusion are usually used for culture, and a positive culture is confirmatory for the diagnosis of cryptococcosis. However, the culture rate is low. Radiologically, chest CT is an excellent method for visualizing pulmonary nodules. Lung metastases typically appear as multiple, peripheral, and rounded nodules scattered throughout both lungs.^[14]^ On the other hand, the radiographic features of PC vary widely. Furthermore, although some authors have reported that immune status influences the radiological features of PC, others disagree. Nodule- or mass-pattern lesions and lesions with a peripheral distribution are the most common features of PC because cryptococcus spreads to the periphery after infection, as demonstrated in our cases. Other main imaging manifestations of PC include patchy shadows, widespread infiltrates, and focal consolidation.^[15,16]^ PC is easily misdiagnosed as metastatic lung cancer because it lacks pathognomonic CT features. The lesions were located peripherally, and most were 1 to 2 cm in diameter. However, the location of the lesions varied. Some studies reported that PC is not associated with a specific lung lobe. However, Hu et al found that PC was most commonly located in the right upper lobe, followed by the right lower lobe, and the left lower lobe,^[17]^ which concurs with that observed in our cases (left lower lobe in case 1 and right upper lobe in case 2). The CT images of our patients showed multiple nodules in the lung peripheries with no calcified foci. In previous studies, PET-CT scans were used to differentiate between benign and malignant masses, but failed to differentiate between PC and lung metastasis.^[18]^ The SUVmax values for PC cases may vary widely from mild to marked uptake (from 0.93 to 13.0),^[19]^ which is consistent with the findings of our cases (3.7 and 1.4, respectively). Serum testing for cryptococcal antigen has also been recommended,^[15]^ but the sensitivity of this antigen in immunosuppressed populations is only 39%.^[20]^ Thus no gold-standard noninvasive diagnostic method exists, and the risk of misdiagnosis remains high. Therefore, definitive diagnoses are usually based on tissue biopsies obtained by percutaneous needle biopsy, trans-bronchial lung biopsy, video-assisted thoracoscopic surgery, or surgical lung biopsy. Although single or multiple discrete, noncalcified nodules are characteristic chest radiographic findings, they are radiologically similar to cancer and warrant lung biopsy. The diagnostic histological finding of PC is encapsulated yeast with a mucicarmine-positive capsule. Histological staining with hematoxylin and eosin (H&E), Grocott or Gomori methenamine silver (GMS), and PAS was used to detect cryptococcus. Furthermore, a wide variety of inflammatory reactions, ranging from well-formed granulomas to minimal inflammation, have been observed in pulmonary cryptococcal infections.^[21]^

Surgical resection is the key to reliable diagnosis and treatment decision-making for fungal lung diseases, and in our patients, surgery was performed for these reasons. Treatments depend on the host immune status, disease extent, symptoms, and whether the disease is disseminated or localized. Oral fluconazole is the drug of choice for symptomatic cryptococcosis without CNS involvement^[22]^ although in immunocompetent patients with PC, an isolated nodule often resolves spontaneously without antifungal treatment. Fluconazole treatment regimens are 200 to 400 mg/day for 3 to 6 months, 6 to 12 months, or >12 months for asymptomatic patients, patients with mild to moderate symptoms, and patients with severe pulmonary infections, respectively. Fluconazole therapy is not required for asymptomatic patients who have undergone surgery; however for those with symptoms who have undergone surgery, fluconazole is administered at a dose of 200 to 400 mg/day for 3 months.^[15]^

In summary, we report the misdiagnosis of PC as lung metastasis in 2 gynecologic cancers. In patients undergoing chemotherapy or radiotherapy, the possibility of PC development should be considered, because the immune system is suppressed. PC is often misdiagnosed as metastatic lung cancer because of its similar clinical manifestations and radiological characteristics. Thus, it should be considered in the differential diagnosis when single or multiple nodules are detected on chest radiography without corresponding tumor marker elevation in gynecological cancer patients with predisposing factors.

There were some limitations of this study, such as the small sample size and a single group of gynecologic cancer patients. The main goal of this case report is to highlight that clinicians should be aware of PC being possibly responsible for lung lesions after treatment of gynecologic cancers.

## Acknowledgments

The authors are grateful to the patients for their contribution to this study.

## Author contributions

**Conceptualization:** Seul Yi Lee, Ki Hyung Kim.

**Resources:** Geewon Lee, Kyung Un Choi.

**Supervision:** Yong Jung Song, Hyung Joon Yoon.

**Writing – original draft:** Seul Yi Lee.

**Writing – review & editing:** Dong Soo Suh, Ki Hyung Kim.
